# Narrative meaning-making at the crossroads between life and death: a qualitative study into contemplating literary texts with advanced cancer patients.

**DOI:** 10.1007/s11764-025-01765-w

**Published:** 2025-02-24

**Authors:** Niels van Poecke, Michael Scherer-Rath, Yvonne Weeseman, Zoë Bood, Nirav Christophe, Henny Dörr, Judith de Vos-Geelen, Aart Beeker, Gerty de Klerk, Eric Bras, Hugo Vlug, Frans Bossink, Frans Savelkoul, Liesbeth M. Timmermans, Esther Helmich, Mirjam A. G. Sprangers, Hanneke W. M. van Laarhoven

**Affiliations:** 1 Department of Medical Oncology, Amsterdam University Medical Center, University of Amsterdam, Amsterdam, The Netherlands; 2 Cancer Center Amsterdam, Cancer Treatment and Quality of Life, Amsterdam, The Netherlands; 3 Department of Philosophy, Theology and Religious Studies, Radboud University Nijmegen, Nijmegen, The Netherlands; 4 iHUB, Rotterdam, The Netherlands; 5 HKU University of the Arts, Utrecht, The Netherlands; 6 Deprtment of Internal Medicine, Division Medical Oncology, GROW – Research Institute for Oncology & Reproduction, Maastricht University Medical Center, Maastricht, The Netherlands; 7 Department of Medical Oncology, Spaarne Gasthuis, Hoofddorp/Haarlem, The Netherlands; 8 Deprtment of Pastoral and Spiritual Care, Amsterdam University Medical Center, Location VUmc, Amsterdam, The Netherlands; 9 Deprtment of Pastoral and Spiritual Care, Amsterdam University Medical Center, Location AMC, Amsterdam, The Netherlands; 10 Department of Spiritual Care, Spaarne Gasthuis, Haarlem/Hoofddorp, The Netherlands; 11 Deprtment of Spiritual Care, Maastricht University Medical Center, Maastricht, The Netherlands; 12 Department of Primary and Community Care, Radboud University Medical Center, Nijmegen, The Netherlands; 13 Amsta Healthcare Organization, Amsterdam, The Netherlands; 14 Department of Medical Psychology, Amsterdam University Medical Center, University of Amsterdam, Amsterdam, The Netherlands

**Keywords:** Contingency experiences, Advanced cancer, Narrative meaning-making, Self-distancing, Literature

## Abstract

**Purpose:**

Advanced cancer patients may perceive their disease as an experience of contingency: a (sudden) disruption of the life-narrative evoking existential concerns such as loss of meaning and identity. The research project *In Search of Stories* aimed to investigate whether and how reading and discussing literary texts assisted participants in integrating their diagnosis as the experience of contingency into their life-narrative.

**Methods:**

This qualitative study reports on interviews with 25 advanced cancer patients, who read a story from a curated collection of 10 literary texts. They discussed the story with a spiritual caregiver, using a reading guide to structure the conversation. The interviews were thematically analyzed using template analysis.

**Results:**

Participants identified with contingency experiences and related existential concerns presented in the texts, including loss of meaning and identity, bodily alienation, and social estrangement. Reading and discussing literary texts also enabled participants to engage in self-regulation; in exploring how to relate to devalued emotions and attitudes, and in further developing their identity post-diagnosis.

**Conclusions:**

A structured reading of literary texts revolving around contingency experiences enables advanced cancer patients to identify with and articulate emotions, attitudes, and existential concerns such as loss of meaning and identity. This allows them to break down barriers in talking about their illness experiences and to transition towards acceptance of their diagnosis as the experience of contingency.

**Implications for Cancer Survivors:**

Reading and discussing literary texts about contingency may support people with advanced cancer to integrate their own experience of contingency into the life-narrative.

**Supplementary Information:**

The online version contains supplementary material available at 10.1007/s11764-025-01765-w.

## Introduction

People with advanced cancer may perceive their disease and its treatment as an experience of contingency. Contingency refers to the idea that everything could have been different. The occurrence of the life event is a possibility, not a necessity [1, 2]. An experience of contingency could be described as a crisis of meaning-making, as people may be challenged to develop a renewed sense of meaning and purpose in life, and to integrate the event into the life-narrative [2–4]. Falling severely ill, then, may have biographical consequences [3, 5], challenging people to reconstruct their life-narrative by integrating the experience of contingency into their identity [6–8].

To support patients with advanced cancer in reconstructing their life-narratives, we developed a theoretical model, which departs from a simple understanding that people make meaning in life in a *narrative* way [3, 9]. People make sense of their experiences, as these unfold throughout time, by constructing stories, which provide their lives with a sense of coherence and purpose. This is a process we define as *narrative meaning-making *[3, 6]. Thus, the life-narrative should be seen as a “form of identity, which both reflects and shapes who a person is” [3].

Events that are disruptive to the life-narrative, like a diagnosis of advanced cancer, consequently require a reconstruction of the life-narrative that transforms the experience of disruption into a renewed sense of coherence and purpose [3, 6]. The latter is a process we refer to as *narrative integration*, and define as the extent of which the disruptive life event is integrated into the life-narrative and becomes a part of one’s (changed) identity [3]. We argue that the process of narrative meaning-making post-diagnosis is not a coping strategy (e.g., to reduce distress) [10, 11], but should be seen as the existential dimension underlying coping and adjustment [3]. That is, it is not distress that evokes existential concerns such as loss of meaning, but the disruption of the life-narrative due to the experience of contingency [3].

Based on this model, we developed *In Search of Stories* (ISOS): a multi-modal intervention for narrative meaning-making in people with advanced cancer [9, 12–15]. As part of this intervention, participants were invited to select, read, and discuss a literary text under the guidance of a spiritual caregiver. We aimed to investigate (1) whether and how participants would identify with experiences of contingency as presented in a collection of carefully selected literary texts, and (2) how the identification with literary texts would enable participants to integrate their diagnosis, as the experience of contingency, into their life-narrative.

## Methods

### Study design

A collection of stories was curated by members of the research team (NC, HD, ZB), as described previously [12]. In brief, the curated collection consisted of 10 literary texts revolving around contingency experiences: sudden disruptions of the unfolding plot, evoking existential questions and concerns in the (lives of) protagonists. Three of the stories were embedded in religious worldviews (*Job*, *Orpheus*, *Yunus*), four of the stories were written in a more contemporary and realist literary style (*The After Days*, *Code Catnip*, *Farewell from Phoebe*, *The Fault in Our Stars*), and the latter three stories were embedded in the genre of Absurdist fiction (*The Ant’s Departure*, *Death of an Old, Old Man*, *Metamorphosis*) (cf. Table 1 for an overview, including authors and synopses).

**Table 1:** Overview of literary stories, authors, and synopses (Synopses are drawn from the curated collection of stories, titled “Wellicht” [“Perhaps”] (2019, in Dutch))

Story	Author	Storyline
*Orpheus*	After Stephen Fry	If Orpheus’ wife gets bitten by a snake, Orpheus is inconsolably sad. He departs to the underworld and tries to get his wife back
*The Ant’s Departure*	Toon Tellegen	The ant has left. The other animals who live in the forest neither know why, nor whereto he has left. They all relate differently to the ant’s departure
*Yunus*	After Ibn Kathir	It is stormy at sea. Yunus ends up in the belly of the whale. He imagines dying
*Job*	Fragments from the Bible verse	Job is a righteous man whose life goes well: he has a lot of property, a large family, and lives in good health. In short time he loses everything. Job revolts
*The After Days*	A.N. Ryst	A son experiences how his parents decline. They all discover that speaking about their experiences, and how their lives are changing, is difficult
*Code Catnip*	Jacques Vriens	If grandpa hears that he will soon die, he and his grandson Stijn don’t really know how to deal with that
*Metamorphosis*	Franz Kafka	When Gregor Samsa wakes up one day, he discovers that his body has changed into the shape of a bug. Despite feeling helpless and clumsy, he decides to think about how to leave his bed and go to work
*The Fault in Our Stars*	John Green	Hazel is incurably ill and in love with Augustus. Despite the situation, they enjoy life. Then, Augustus is the messenger of bad news
*Death of an Old Old Man*	Roald Dahl	After his plane crashes, a pilot fights for his life. Reality and delusion are intertwined
*Farewell from Phoebe*	Vonne van der Meer	A woman gives birth to her death child. Her struggle is tough and lonely

Participants of the ISOS intervention could either read the stories from a paper booklet, or read and/or listen to the stories on an accompanying website, including short audiovisual summaries. A reading guide was developed to support participants in the process of identifying with the literary texts, and consisted of three consecutive stages: close reading, recognition, and connection [12]. These stages were related to three dimensions of narrative identification: (1) a cognitive-linguistic dimension, referring to the identification with the textual level of the literary story (e.g., words, concepts, figures of speech); (2) a psychological dimension, referring to the identification with the participant’s life-narrative [6, 16]; and (3) an existential dimension, referring to the process of connecting story aspects to participants’ overarching worldview [3].

The reading guide functioned as an interview protocol, the questions of which enabled participants to move from a descriptive discussion of the meaning of the text towards a more reflective discussion of the meaning of the text in the context of their own lives marked by living-with advanced cancer. Discussions started with getting acquainted and with a close reading of the text, after which the spiritual caregiver would ask questions (e.g., “Who do you hear?,” “What do you see?”) to assist participants in reading carefully and, accordingly, in creating a mental understanding of the text. During stage two (recognition), the spiritual caregiver would ask questions to investigate whether and how participants recognized themselves in subjects and situations presented in the fictional stories. Questions were asked, such as “In whom do I recognize myself?” and “Which event do I recognize?” Stage three (connection), finally, focused on reflection and consisted of questions that were, like in stage one, formulated in the second person (e.g., “How does the story affect you?”). It aimed to prompt thoughts, ideas, and emotional responses in participants, investigating what the story would personally mean for them [12].

### Inclusion and procedure

Eligible participants were ≥ 18 years of age; had a diagnosis of metastatic/inoperable cancer; had sufficient mental, verbal, and physical capacity to participate; and had a life expectancy beyond the planned duration of the intervention (≥ 3 months). A degree of diversity according to gender, tumor type, and age was aimed for (cf. Table 3 for participant characteristics). Patients who met the inclusion criteria were recruited from the outpatient departments of Medical Oncology through their attending oncologist or nurse at three university medical centers in the Netherlands—Academic Medical Centre (AMC), Free University Medical Center, and Maastricht UMC—and one regional hospital: Spaarne Gasthuis (Haarlem/Hoofddorp, the Netherlands). After written informed consent, participants received the curated collection of literary texts, read the stories at home, and selected one story for a discussion with a spiritual caregiver in the hospital. The medical ethics review committee of the AMC decided that the Medical Research Involving Human Subjects Act did not apply to the study (reference number: W20_436 # 20.483).

### Data analysis

All conversations between participants and spiritual caregivers were audio recorded on an encrypted device (Philips PocketMemo DPM-8000), and lasted between 32 and 78 min. The audio recordings were transcribed *ad verbatim*, including observations of silences and both verbal and physical expressions of emotions. The transcriptions were stored on a secured hospital drive (location AMC), and uploaded to qualitative software analysis program MaxQDA 2022 (version 22.1.1). For the systematic analysis of the conversations between participants and spiritual caregivers, we used template analysis, which is a type of thematic analysis useful to demarcate theory-informed themes within the empirical data [17–19]. Based on a pilot study [12], an initial template was used. After reading and rereading the interview transcripts, the initial template was further modified and refined by NvP (cf. Table 2). Intercoder reliability was secured as NvP iteratively discussed findings of the data analysis with project group members HvL and MS-R.

**Table 2:** Template analysis: overview of (sub)themes emerging from data analysis conducted for the present study

Themes	Subthemes
1. Selection of story	1.1. The Ant’s Departure 1.2. Code Catnip 1.3. Death of an Old Old Man 1.4. Farewell from Phoebe 1.5. The Fault in Our Stars 1.6. Metamorphosis 1.7. Orpheus 1.8. Yunus
2. Narrative identification	2.1. Character traits protagonist 2.2. Situations 2.3. Emotions 2.4. Interpretations of fate 2.5. Social environment: 2.5.1. Normative expectations 2.5.2. Feelings of love and affection from/towards (significant) others 2.6. Non-identification
3. Experiences of contingency	3.1. Existential concerns: 3.1.1. Loss of autonomy 3.1.2. Loss of identity 3.1.3. Loss of life-goals 3.1.4. Loss of future roles and expectations 3.1.5. Bodily alienation 3.1.6. Social estrangement 3.2. Non-cancer related contingency experiences
4. Concepts of meaning	4.1. Instrumental life goals 4.2. Love and affection from/towards (significant) others
5. Sources of meaning	5.1. Christian 5.2. Secular 5.3. Other religious
6. Self-regulatory aspects	6.2. Regaining autonomy over post-illness identity: 6.2.1. Exploring impending death 6.2.3. Exploring future scenarios related to death and dying 6.2.4. Exploring articulation of existential concerns 6.2.5. Exploring ‘fierce’ emotions 6.2.6. Broadening perspectives of the self 6.2.7. Strengthening self-identity

Compared to the template that was generated for the pilot study (cf. “Data analysis”), the template for the current study consisted of one additional theme: Theme 6, “Self-regulatory aspects of reading and discussing a literary text with a spiritual caregiver.” Other themes were modified and refined. The theme “narrative identification” initially included the subthemes “subjects” and “situations.” However, after analysis of the data for the present study, it included the subthemes: “character traits protagonist,” “situations,” “emotions,” “interpretations of fate,” “social environment,” and “non-identification.” Theme 3, “Experiences of contingency,” initially consisted of the subthemes “loss of life goals,” “impending death,” and “uncertainty,” but for the current study included “existential concerns,” “loss of identity,” “loss of life goals,” “loss of future roles and expectations,” "bodily alieanation," "social estrangement," and “non-cancer-related contingency experiences.” Theme 4, “Concepts of meaning,” initially included “fate,” “life goals,” “quality of life,” and “death,” but now included “instrumental life goals” and “love and affection from/towards significant others.” Themes 1 and 5 remained, except that theme 1 included more literary texts compared to the pilot study, as (more) participants selected a wider diversity of stories

## Results

### Patients and their worldviews

Twenty-six participants were included [14]. One participant (P14) died shortly after giving written informed consent, leaving twenty-five participants available for analysis. The majority of participants was female (*n* = 18), and most participants had religious worldviews (*n* = 19). These included Christian worldviews (*n* = 9), a Jewish worldview (n = 1), or other religious worldviews (*n* = 9), such as Buddhism. The age of participants ranged from 28 to 80 (*m* = 58.3 years). The majority of participants (*n* = 19) had academic degrees; others had backgrounds in (pre-)vocational educational (*n* = 6). Participants had been diagnosed with a diversity of metastatic diseases, including cervical, breast, colon, bone, and esophageal cancers (cf. Table 3 for a complete overview).

**Table 3:** Overview of participants in the project *In Search of Stories* (ISOS)

Patient	Gender	Age	Tumor type	Selected story	Educational level	Worldview
P1	Female	43	Cervical	*Phoebe*	Academic	Christian
P2	Female	50	Cervical	*Yunus*	Vocational	Secular
P3	Female	49	Cervical	*Ant’s Departure*	Uni Applied Sciences	Other religious
P4	Female	68	Cholangio	*Phoebe*	Uni Applied Sciences	Christian
P5	Female	56	Bile duct	*Yunus*	Uni Applied Sciences	Secular
P6	Female	28	Soft tissue sarcoma	*Yunus*	Academic	Christian
P7	Female	76	Gastric	*Code Catnip*	Uni Applied Sciences	Secular
P8	Female	59	Colon	*Orpheus*	Uni Applied Sciences	Other religious
P9	Female	60	Esophageal	*Fault In Our Stars*	Uni Applied Sciences	Other religious
P10	Male	65	Esophageal	*Code Catnip*	Vocational	Secular
P11	Female	48	Breast	*Metamorphosis*	Vocational	Secular
P12	Male	73	Esophageal	*Ruth & Boaz*	Uni Applied Sciences	Jewish
P13	Male	80	Esophageal	*Orpheus*	Pre-vocational education	Christian
P15	Female	55	Pancreatic	*Orpheus*	Academic	Other religious
P16	Male	65	Pancreatic	*Veteran*	Pre-vocational education	Secular
P17	Female	61	Breast	*Fault In Our Stars*	Uni Applied Sciences	Christian
P18	Male	50	Gastric	*Ant’s Departure*	Uni Applied Sciences	Secular
P19	Female	68	Rectal	*Ant’s Departure*	Uni Applied Sciences	Christian
P20	Female	49	Breast	*Code Catnip*	Academic	Secular
P21	Female	65	Neck	*Metamorphosis*	Uni Applied Sciences	Christian
P22	Female	67	Breast	*Code Catnip*	Academic	Secular
P23	Female	54	Breast	*Metamorphosis*	Academic	Other religious
P24	Male	71	Endocrine	*Code Catnip*	Uni Applied Sciences	Christian
P25	Female	46	Breast	*Fault In Our Stars*	Vocational	Christian
P26	Male	51	Bone	*Ant’s Departure*	Academic	Secular

### Selected stories

The majority of participants (72%) selected non-religious stories, irrespective of their worldview (Table 2—Subthemes 1; Table 3). Of the participants who selected religious stories, the majority (57%) had a religious worldview. Within the category of non-religious stories, 55% of the participants selected realist stories, others selected stories embedded in the genre of Absurdist fiction. One participant selected a story from outside the curated collection: the story of “Ruth and Boaz” from the Hebrew Bible. Two stories from the collection were not selected: the story of *Job* from the Bible and *The After Days* by Dutch author A.N. Ryst. The interview transcripts did not contain data on why these two stories were selected by neither of the participants.

### Narrative identification and experiences of contingency

Participants identified with the following aspects of the literary texts (Table 2—Subthemes 2): character traits of protagonists, situations and emotions narrated in the stories, protagonists’ experiences of fate unfolding in life, protagonists’ experiences of normativity imposed by (significant) others, and feelings of love and affection from and towards loved ones. Some participants were unable to identify with any of the literary texts, thus also non-identification emerged as a theme. Furthermore, participants identified with existential concerns narrated in the literary texts, including bodily alienation, social estrangement, and loss of identity, life goals, and future expectations (Table 2—Subthemes 3); as well as with concepts of meaning (Table 2—Subthemes 4), such as instrumental life goals (e.g., traveling) and sharing/receiving feelings of love and affection with/from significant others. Below, we discuss our findings in more detail.

#### Character traits

Participants with secular worldviews mostly identified with protagonists who performed independent, or socially resistant behavior. Being independent/resistant was interpreted as a life value that could counteract the experience of being framed as a ‘patient’ and having to show patient-associated behavior in social life, such as behaving prudently and performing vulnerability. In some of the stories, such as *Code Catnip*, normative expectations imposed by significant others were counteracted by protagonists through the performance of resistant behavior, as well as by using dark humor to shock loved ones. Participants identified with such an attitude of social resistance, as it enabled them to explore how to maintain autonomy within the situation of being framed as a ‘patient’ and, thus, to experience of loss of autonomy (Supplementary Table S4: quote 1).

Participants who selected religious stories emphasized the ability of protagonists to act as a “fighter” (P5) or “warrior” (P6) in opposition to their fate. Thus, they valued the attitude of protagonists in religious stories, such as the stories about Yunus and Orpheus, who searched for possibilities to confront their fate, regardless of its invincibility. Participants acknowledged such character traits, as they identified with the promise of finding redemption in their life; i.e., finding some kind of material and/or spiritual “reward” for confronting their fate heroically (Supplementary Table S4: quotes 6–8).

#### Situations and emotions

Reading and discussing the stories triggered autobiographical memories of (social) situations in participants, including those related to loss of relatives due to cancer and suicide, or to giving birth prematurely before their illness (Supplementary Table S4: quotes 2–4). Also, reading and discussing a literary text triggered memories of situations marked by living-with cancer, ranging from hearing the diagnosis for the first time to the impact this had on relatives, receiving support from relatives, experiencing social estrangement, experiencing a loss of autonomy and dissociation from the body, and facing physical and financial constraints due to cancer and its treatment (Supplementary Table S4: quotes 9, 17, 21). 

Furthermore, reading and discussing the stories with a spiritual caregiver elicited emotions in participants, ranging from ‘positive’ emotions, such as empathy, love, happiness, and relief, to ‘negative’ emotions such as sadness, anxiety, despair, grief, and anger. Participants identified with these emotions *in* the stories, and experienced emotions in and through the process of reading and discussing the texts together with a spiritual caregiver. Regarding the latter, P3, for example, explained that immersing herself in *The Ant’s Departure* by Dutch author Toon Tellegen enabled her to experience happiness, particularly after reading about how some of the animals in the story were not merely sad about or denying the sudden disappearance of the ant (protagonist), but were actively acknowledging it. This reflected her wish of how her relatives should ideally react to her passing away; i.e., by openly processing their grief, rather than by closing themselves off or by denying it (Supplementary Table S4: quote 5).

#### Interpretations of fate

Participants interpreted their diagnosis of advanced cancer in different ways: while most perceived it as an experience of contingency; i.e., as a life event that constitutes the randomness of life, some perceived it as a life event that occurred necessarily. The latter interpretation strategy was exemplified by P5, P6, P15, and P23 (Supplementary Table S4: quotes 6–9). In reference to *Orpheus*, P15, for instance, explained that the diagnosis was “meant to be.” P5, relating to *Yunus*, perceived her diagnosis as the consequence of the necessity to reduce overpopulation, or, to “make the ship less heavy,” metaphorically referencing the selected story. P6 believed that her “fate was predetermined,” as she compared her cancer to Yunus being swallowed by a whale, representing a force from the beyond. Analysis, moreover, reveals that these participants frequently used quest metaphors to discuss their illness, such as “fighting cancer despite not conquering it” (P5), as well as that they perceived their disease progression as a “journey” (P5) that could be spiritually rewarding. P6, for instance, drew parallels between Yunus’ salvation by God and her own cancer diagnosis, which brought greater awareness of the “good things in life,” including a “healthier lifestyle” (Supplementary Table S4: quote 8). P23, finally, retrospectively perceived the unfolding of fate in her life as a necessarily disastrous situation that was evoked not by an external force, but—somewhat tragically—by her own behavior. Due to structural financial misconduct in the past, she developed chronic stress and this, in her perception, eventually caused cancer. P23 experienced it as a tragic situation of being “trapped” by fate, similar to Gregor Samsa’s situation in Kafka’s story *Metamorphosis*, who wakes up one day and discovers that he has changed into a monstrous bug lying on his bed, not being able to move. As P23 summarized: “I have created a situation myself for which I am responsible myself” (Supplementary Table S4: quote 9).

The majority of participants, as said, interpreted their fate as the experience of contingency: an unexpected life event that shattered their life-narrative. Some participants selected a literary text because it recalled contingency experiences in their lives before cancer, including marital break-up (P3), experiencing a miscarriage (P4), being unable to have children (P1), and losing lost ones to cancer (P7/P22) or suicide (P10). Most participants, however, selected a text as they connected it to their diagnosis of advanced cancer as the experience of contingency. This is evident from participants’ accounts on how their diagnosis disrupted their (ultimate) life goals and future expectations and roles, such as experiencing infertility from cancer treatment, evolving into the inability to be a parent; dissolving romantic relationships due to breast surgery, affecting one’s self-image and self-esteem; and loss of work (life) due to reduced energy and concentration as a consequence of cancer treatment, affecting a loss of one’s work-related identity (Supplementary Table S4: quotes 10–12, respectively). Additionally, the perception of the cancer diagnosis as the experience of contingency is reflected in discussions of existential concerns, such as experiencing alienation from the body and the social environment, as well as in discussions about loss of meaning, autonomy, identity, or “decorum”—as P1 and P8 explained (Supplementary Table S4: quotes 13, 14).

#### Normative expectations around behavior and emotions

Participants identified with scenes in the literary texts where protagonists felt “alone with their story” (P9). This was because significant others, in participants’ perceptions, failed to discuss their illness experiences meaningfully, for instance because they purposefully silenced it or, when sharing concerns, by using “empty euphemisms” or by producing “word salads” (P4). Participants also identified with experiences of loneliness enacted in the stories, as these were tied to the taboo subject of (not) discussing incurable disease and mortality; as well as with the imposition of normative behavior, leading to what P3 called “emotional pollution.” P4 illustrated this emic concept with a scene from *Farewell from Phoebe*, where a healthcare worker projects his own emotions onto the protagonist, leaving her unable to feel and express herself authentically (Supplementary Table S4: quote 15). Other participants similarly shared how such situations resulted in their emotions to be “taken away” by others (P4), to be “tucked away by themselves” (P23), or allowing them only to “feel in slow motion” (P9). Also, they discussed their struggles in finding a balance between being both a patient and being a person behind the patient. P24 highlighted this tension through scenes from *Code Catnip*, where a terminally ill protagonist tries to enjoy time with his grandchild, while being confronted with constraining expectations imposed by loved ones, framing him as a vulnerable patient (Supplementary Table S4: quote 16). Finally, participants noted how they began managing relatives’ emotions, rather than taking care of their own emotions, resulting in experiences of social estrangement and emotional dissociation, as P1 explained by referencing *Farewell from Phoebe* (Supplementary Table S4: quote 17).

#### Non-identification

The inability to identify with (any) elements within the literary texts also emerged as a theme. P12, for instance, decided to select a text himself—the story about “Ruth and Boaz” from the Hebrew Bible—as the stories in the curated collection were “too depressing” as they all “ended on the graveyard.” The self-selected story matched his current life goal of “giving something extra to people who live in poverty.” Similarly, other participants explained that they could not resonate with most of the stories in the collection as they perceived them as reflecting “merely sadness and grief” (P17). Eventually, they selected a story in which humor, having a positive attitude, and the love between main characters were dominant themes, such as in *Code Catnip* and *The Fault in Our Stars*. P8 adopted a different strategy and decided to rewrite the story of *Orpheus* herself. She did this so that she could transpose her own situation of experiencing a gradual loss of identity or “decorum” onto the mythological story and to be able “to give her life back some decorum, namely by adding Gods to the story, who respond, who give strength.”

### Concepts of meaning

#### Love and affection from/towards (significant) others

Participants did not only identify with situations in the literary texts of being socially framed as a ‘patient,’ but also with situations enacted in the stories where protagonists experienced loving relationships and connectivity with (significant) others, as P15 explained (Supplementary Table S4: quote 18). Alongside love and social connectivity, patients also referred to instrumental life goals as concepts of meaning, such as traveling or going on short trips, similar to how the protagonist in *Code Catnip* decides to experience ‘reckless’ adventures with his grandson, despite warnings by his loved ones. Said instrumental life goals, however, were minor themes compared to love and connectivity, which appeared to be major themes in the narratives of participants.

### Self-regulatory aspects of reading and discussing a literary text

Our study also showed how engaging in narrative identification enabled participants to make sense of their current life circumstances marked by living-with advanced disease, and thus to re-engage in narrative meaning-making and even to work on the integration of cancer, as the experience of contingency, into the life-narrative. Below, we reflect on the self-regulatory aspects of selecting, reading, and discussing a literary text under the guidance of a spiritual caregiver (Table 2—Subthemes 6).

#### Regaining autonomy

Reading and discussing literature enabled participants to regain a sense of autonomy over the construction of their identity. Reading and discussing a literary text, more specifically, assisted them in exploring impending death and related emotions and future scenarios, such as the grieving of their relatives after their death (Supplementary Table S4: quote 19). In this context, participants frequently addressed the importance of figures of speech, including metaphor and allegory. P15, for example, explained that the story of *Orpheus* enabled her to explore impending death by the appropriation of metaphors such as “descending to, “walking in,” and “climbing up from” the “underworld,” i.e., by visualizing herself in scenes from Orpheus and thus by connecting it to her current path of moving towards end of life (Supplementary Table S4: quote 20). P9 summarized the process of exploring future scenes and scenarios related to death and the process of dying metaphorically herself as the ability of literature “to see the road a little bit better.” That is, engaging with imagery evoked by figures of speech used in the literary texts enabled her, but also other participants, “to come closer to [their] own story” (P1). Hence, to move towards acknowledgment of their impending death and to explore the process of dying. Related to this, participants framed the process of reading and discussing the literary text as a “key moment” or “tipping point” (P3) in thinking about death. Moreover, it allowed them to explore and articulate their preferred process of dying and, for instance, to reflect on how they relate to euthanasia and/or to leaving loved ones behind with dignity (P24). Finally, appropriating metaphors and allegories, assisted participants in sharing illness experiences with others within their social environment. This was particularly helpful in sharing difficult to articulate existential concerns, such as experiencing bodily alienation. As P11 explained, he would not have had the opportunity to visualize himself as “a bug that lies on its back,” and to refer to himself while discussing his illness experiences with others, without having read Kafka’s story (Supplementary Table S4: quote 21).

#### Exploring “fierce” emotions

Participants used the conversation with the spiritual caregiver as an opportunity to express emotions both verbally and physically. Regarding the latter, they frequently cried, but predominantly laughed about their stories describing their existential concerns (Supplementary Table S4: quotes 13, 20, 21–24). Regarding the former, participants explained that reading and discussing a literary text enabled them to “embody the intruding sentences” (P9), as it prompted them to “explore the emotional trigger” (P22)—or, more precisely, “to explore how to relate to emotions” (P23). That is, reading and discussing a literary text about how protagonists were being challenged by the experience of contingency assisted them in processing “fierce” emotions (P22), including sadness, anxiety, anger, grief, and despair. Exemplary is how P22 referred to *Code Catnip*, a story in which the protagonist first deliberately refuses to be sad, yet gradually moves towards acceptance of his fate. Similarly, P22 explained that reading the text and, subsequently, discussing it with the spiritual caregiver “broke down barriers” (P22) in allowing “fierce” emotions into her life. This opened up ways of imagining *and* articulating impending death and the process of dying and, thus, to move towards acceptance of the diagnosis of cancer as the experience of contingency (Supplementary Table S4: quotes 25, 26).

#### Broadening perspectives

Participants discussed the potential of literature to offer “material to compare” (P12), or to look at one’s life from a “different perspective” (P21); i.e., to explore different options in relating to one’s current life circumstances marked by contingency and narrative disruption. P26, for example, explained that reading *The Ant’s Departure* helped him to consider different options in dealing with his diagnosis. Initially, after receiving the diagnosis, he wanted to “live at the top of his game.” By this, he meant that he wanted to take care of his family somewhat heroically. Gradually, however, he discovered that he felt too sad to be able to do so—similar to one of the protagonists, the squirrel, in Toon Tellegen’s fable. After reading and contemplating the story with the spiritual caregiver, he realized that he could not “save his family from grief” and that living at the top of his game would, in fact, “only increase the sadness.” Therefore, he decided to be like the ant (the main protagonist in the story), who left a note to fellow animals after his departure, saying “The ant, that is me.” For P26, the quote signifies that it is enough to ‘simply’ be himself, functioning as a form of self-affirmation (Supplementary Table S4: quote 27).

#### Strengthening self-identity

Finally, reading and discussing a literary text supported participants in (re-)discovering desired aspects of their identity; i.e., to use literature as a “mirror” (P15) of the self. One example is how P4 shared with the spiritual caregiver that reading a fragment from *Farewell from Phoebe* triggered autobiographical memories in her of having had an abortion earlier in life, as well as of being hospitalized at the Intensive Care Unit shortly after being diagnosed with advanced cancer. Similar to the protagonist in the story, during both situations, P4 felt muted as a consequence of others inflecting her emotions. Reading and discussing the story reminded her of how she desired to be “less conflict-averse,” a character trait that she had since childhood but now aimed to change after reflecting on her experiences of being constrained from expressing herself authentically (Supplementary Table S4: quote 28). That is, identifying with the scene from *Farewell from Phoebe* enabled her to regain autonomy in making (crucial) decisions related to her cancer treatment. Interestingly, connecting with a desired self (a person who no longer avoids conflicts) was facilitated by comparing her own behavior with both the behavior of the protagonist (Phoebe’s husband Otto) in the story and the behavior of her own husband. During her time at the Intensive Care Unit, her husband behaved very confidently; unlike Otto, who is conflict-averse and therefore does not take good care of his wife. Using the scene from the literary text as a mirror situation, and by transposing herself onto the role of her own husband, P4 found strength to change said aspect of her identity.

## Discussion

Our aim was to investigate whether and how participants of the project *In Search of Stories* (ISOS) would identify with experiences of contingency as presented in a curated collection of literary texts, and how this would facilitate narrative integration post-diagnosis. We found that reading literary texts allowed participants to explore their emotional and existential responses to confronting cancer as the experience of contingency, although some of the participants did not perceive their fate as a contingent life event, but as a life event that evolved into their lives necessarily.

Figures of speech, such as metaphor and allegory, were highlighted as key instruments, enabling participants to visualize current situations and future scenarios, such as their impending death and the grieving process of their relatives. Through language prompting imagery, participants were afforded to project such situations and scenarios onto the literary scenes. This fostered their ability to perceive and evaluate their own lives through a narrative-aesthetic lens; i.e., by adopting what is known as the “aesthetic attitude” [9, 20].

Scholars within the humanities have argued that experiencing reality by applying the aesthetic attitude offers “an affordance of perceptual engagement but yet non-action, which opens up possibilities for using our minds – and brains – in ways we do not in our regular, practically engaged modes of perception” [20, 21]. By experiencing reality aesthetically, one is enabled to temporarily set aside the more habitual or practical way of experiencing reality and, accordingly, to explore new possibilities in relating to reality. Experiencing reality in the aesthetic mode thus requires a degree of distancing from our ‘default’ way of experiencing, such as seeing a chair not as an object to sit or stand on, but as an object that can be appreciated ‘merely’ for its beauty [34].

Translating this to our work of designing supportive care interventions for patients with cancer, this hypothetically implies that by engaging with art, patients are enabled to foster a degree of self-distancing [9]. That is, the ability to perceive and evaluate one’s live through a third-person perspective and, hence, to temporarily detach oneself from the “patient role” [22] so as to develop new ways of relating to the shattering experience of disease-evoked contingency [4, 7, 23]. Findings from our study support this hypothesis, particularly in cases when participants explained how they appropriated metaphors from the texts and how this enabled them to “come closer to their own stories,” or in cases when engaging with a literary story offered participants “material to compare.” Thus, by looking at their own lives through particular literary scenes, this allowed participants of our intervention to develop a different mode—and accompanying mood—in relating to their diagnosis of advanced cancer as the experience of contingency.

Self-distancing, alongside self-transcendence, could be perceived as a fundamental human quality, as it provides people with a sense of ownership in (re-)constructing their life stories [4, 24]. Self-distancing, however, may be difficult for those living-with severe illness, whose personal narratives are to some degree shaped by societal discourses [25–27]. Participants of our study resonated with experiences of protagonists in the literary texts who, as patients, were socially framed in ways that muted and marginalized them. This social framing prompted compliance with devalued emotions, such as anger, despair, and grief, as well as with personal attitudes, such as being resistant to existing norms and values, by behaving ‘recklessly,’ or by applying dark humor so as to perform an aesthetics of shock. Participants noted that forms of social resistance were often misunderstood or suppressed by those around them. Thus, indeed, patients with cancer may not only be “victims of their fate, but [also] of the story with which they have framed it.” [4]

Previous sociological research on cancer survivorship, in fact, shows that certain attitudes, like resilience, positivity, and behaving heroically, are socially valued, while others, such as grief, sadness, anger, or resistance, are devalued [26, 27]. This type of social dynamic shapes peoples’ illness experiences and their personal life-narratives. Narrative-aesthetic interventions, such as *In Search of Stories*, assist participants in reflecting on the process of being socially framed, a process which may result in the concealing of particular emotional states and personal attitudes. Concurrently, said intervention supported participants in reconnecting with socially devalued emotions and attitudes. Exploring and articulating devalued emotions, in fact, appeared to be crucial for allowing (thinking about) impending death and the process of dying into their lives. Such emotions were perceived as “fierce” by participants and members of their social environment, and were therefore mostly obscured. Yet, reconnecting with those emotions during the process of reading and discussing a literary text supported participants in moving towards acceptance of their diagnosis, and thus in integrating cancer as the experience of contingency into their life-narrative.

The exploration of emotional and existential experiences was expressed both verbally and physically by participants, the latter through crying and laughter. According to the philosopher Helmuth Plessner, these are (opposite) bodily responses that occur in response to “boundary situations” where language falls short [28]. Participants frequently cried, but predominantly laughed when discussing story fragments that confronted them with the limits of their existence. This reflects the embodied nature of making sense of ‘absurdist’ existential concerns, such as experiencing alienation or dissociation from the body or the social environment. Reading and discussing literature with a spiritual caregiver, however, enabled participants to make sense of these existential concerns: physically, through crying and laughing, but also non-physically, i.e., by applying language. Metaphors appeared to be crucial here, too, as they enabled participants to find words for their experiences and to share their existential concerns with others in their social environment. This demonstrates that discussing a literary text may facilitate bodily forms of meaning-making, but also provides language to articulate these experiences and thus to support in narrative meaning-making.

### Strengths and limitations of the study

Despite increasing awareness of the need for psychosocial care for advanced cancer patients, their existential concerns are frequently unaddressed [4, 29, 30]. As innovations in cancer treatment, like immunotherapy and targeted therapy, prolong life, patients must find ways to live with incurable disease endurably [31, 32]. Hence, support for dealing with existential concerns is increasingly necessary. In this study, we demonstrated that engaging patients in discussions about literary texts on contingency experiences allowed them to connect with generally neglected emotional experiences and existential concerns. A strength of the ISOS intervention, thus, was its ability to encourage participants to adopt a narrative-aesthetic attitude on their lives marked by living-with incurable disease, which helped them to counterbalance the normative expectations imposed upon them and to regain autonomy over the construction of their own life-narrative.

A limitation of the study was the use of a reading guide in practice. The guide aimed to navigate discussions from cognitive-linguistic to psychological and existential dimensions of narrative identification. Discussions with the spiritual caregiver revolved around how participants identified with existential concerns presented in the texts, as well as that they triggered stories on how the diagnosis of advanced cancer affected participants existentially, as in experiencing loss of identity. Discussions, however, mostly centered on psychological aspects of reading literature, where participants focused on how engaging in the intervention helped them to regulate their emotions and to reconfigure their life-narrative. This may be due to the fact that spiritual caregivers facilitating the discussions are not used to working with protocolled guides [33]. More experience with the reading guide that was developed for this study may be necessary to fully implement this approach.

As discussed, a few participants did not perceive their fate as a possibility (i.e., as a contingent life event), but as a necessity, or predetermined life event. As argued elsewhere [3], interpreting one’s fate as an experience of contingency is a consequence of modernity. Due to processes of secularization and individualization, more traditional master narratives have lost their influence, making people responsible themselves for the construction of their life-narratives [3, 25]. Nevertheless, as our empirical findings suggest, other interpretation strategies may persist in modern societies, based on and offering people diagnosed with (advanced) cancer a narrative framework through which they can make sense of their illness experiences. This may be a different narrative framework as our theoretical model suggests. In designing follow-up interventions, in co-creation with patient groups with a diversity of cultural and socio-economic backgrounds, we will take this into account.

A final limitation was the unequal distribution of educational levels among participants, with the majority having academic degrees. This likely influenced their preference for reading and their ability to reflect on literary texts. For the design of follow-up interventions, it could be helpful, for instance, to also work with graphic novels and/or comics, containing imagery that may foster participants’ aesthetic attitudes [34]. Nevertheless, this study highlights that participants with a background in vocational education predominantly engaged with stories featuring abstract elements, such as those from the genre of Absurdist fiction. Although previous research in sociology suggests that the aesthetic attitude is more accessible to those trained in it through family socialization and education [35–38], our findings indicate that also participants with (pre-)vocational educational backgrounds developed an aesthetic attitude, allowing them to work on reconfiguring their life-narratives.

## Conclusions

A structured reading of literary texts revolving around contingency experiences supports people with advanced cancer to explore alternative modes of relating to their own cancer-evoked experiences of contingency. The discussion of devalued emotions, attitudes, and existential concerns supports patients in visualizing, exploring, and articulating their relationship with their impending death and their process of dying, so that they are enabled to move towards acceptance of their diagnosis of cancer and, thus, to work on integrating the experience of contingency into their life-narrative.

## Supplementary Information

Below is the link to the electronic supplementary material.

Supplementary file1 (DOCX 37 KB)

## Data Availability

Data is provided within the manuscript or supplementary information files.
