# Supplementary material for: Narrative meaning-making at the crossroads between life and death: a qualitative study into contemplating literary texts with advanced cancer patients
Source: J Cancer Surviv. 2025 Feb 24;20(4):1658–69. doi: 10.1007/s11764-025-01765-w (PMC13375803; doi:10.1007/s11764-025-01765-w)
Supplement: Supplementary file 1 — Supplementary file1 (DOCX 37 KB) [file 11764_2025_1765_MOESM1_ESM.docx]

**Supplementary table 4** - Overview of participants’ quotes

| **Number** | **Participant** | **Theme** | **Quote** |
| --- | --- | --- | --- |
| 1 | P10 | Identification with character traits | P10: A very good friend of mine, with whom I go on motorcycle rides, he really thinks ‘Well, riding motorcycles, that’s not going to happen for a while… I can put the bike in his garage. (…) Then I thought ‘Come on!’  SCer: What kind of feeling did that give you?  P10: Well, it was something like ‘Wait a minute, goodbye? Yes, but it’s not that far yet!’. I knew that for sure. (…) But I still remember, then we went for a ride, it was a beautiful day, I’ll never forget it… And then I sat with a tear behind my helmet thinking ‘Damn, this won’t be the last time that I get to experience this. And then another voice comes along and says: ‘So enjoy it to the fullest!’ [laughing].  SCer: But that tear and that question, that was actually because of that friend, because of his remark?  P10: Yes, more because of the attitude of ‘Let’s just put [the motorcycle] away, because nothing is going to happen for the time being’. I thought: ‘I’m not going to let myself be put away!’  (…) I wasn’t irritated by it or anything. I actually found it funny, because that actually strengthens my feeling, the contradictory nature of ‘that’s not going to happen!’.  SCer: Rebellion...  P10: Yes, yes. And I really saw that in parallel with that grandpa [protagonist *Code Catnip*]. |
| 2 | P4 | Identification with situations | P4: Oh yeah, that's why it [the story, *Farewell from Phoebe*] resonates with me so much...  SCer: [W]hat resonates with you, in your own life story?  P4: Well, from the past. I had a miscarriage myself when I was 4 months pregnant. I was very young then. It was a wanted child, it wasn't a surprise or anything. |
| 3 | P26 | Identification with situations | P26: Yes, maybe... Yes, that I am more like the hedgehog than the squirrel. The squirrel, he also adores everything about himself a little bit, right? Everything he had written down... I also have a little book from my grandfather [who died from cancer, NvP] in which he left messages for his children and grandchildren. Little lines, pieces of 5 lines or so. Yes, very personal. I don't know why I got it. I think my mother got it from grandma. And my mother gave it to me. A little notepad with my grandfather's handwriting. |
| 4 | P10 | Identification with situations | P10: My sister-in-law... Yes, two years ago, she was depressed for a long time, she jumped from a flat. We kind of saw that coming, yes, not that she would actually do this, but she was depressed and in treatment. She was with us a lot. And I always joked with her. Everyone still says that, the whole family, about the way you treated her, you could say anything to her and joke around. (…) That kind of direct humor [similar to the protagonist in *Code Catnip*, NvP]. How did I come up with that? Well, she jumped from the flat, drama, while two months earlier her brother, my wife's other brother, had hanged himself. |
| 5 | P3 | Identification with emotions | P3: Yes. It makes me... This makes me totally happy every time... And that was the difference with all the other stories, where you always thought ‘Yes, okay, this is a description of how it is, and you know it too, yes of course there is grief’. But this is the story [*The Ant’s Departure*], it just made me happy. And then I think that is what I want, I want to be happy, too. (...) The squirrel is my daughter, very clearly, it suits her so well. Just... let me say what makes them different from the others, who try to revive the ant in the way they deal with their grief. It is not denial... The ant is gone, but what do we have to do to keep the ant with us? The cricket just denies it, it is not true at all, he is not gone. That does not suit anyone in my inner circle. We are too open and honest with each other for that. But I do understand that both my children will try to hold on to me in their own particular way. |
| 6 | P5 | Identification with fate | P5: Yunus then goes on a journey, and when I draw parallels like that, I think, well, it's all a journey. And on that journey you encounter things. And this is what Yunus encountered, a mountain, which was almost impossible to conquer, but he still managed it. (…) And in such a way that he does not come out of the battle completely unscathed, but he does take on the battle and he does it with a belief in something better. (…) And I also draw that parallel with my own life. |
| 7 | P5 | Identification with fate | P5: Yes, I do recognize myself in Yunus, yes, definitely. (…) And especially in the beginning, when he gets the message three times, that yes, actually, a certain death. Because he goes into the sea, so actually it is over and done with. (…) And then he comes back again, and then heal again... Or at least be able to get to the point where he can pick up his life again. So with a long beard and a burned body, but it is possible. (…)  SC: Yes. Because you were actually also told in the same way: ‘There is the sea!’?  P5: Yes, actually yes. (…) You can swim around for another 6 months, but then it just stops. So yes, in that way I do see the parallels, yes. |
| 8 | P6 | Identification with fate | P6: Yes, but maybe also, ultimately, with a good intention from God, to save him from that storm. (…) And that is also how I sometimes look at my cancer (…) how my lifestyle has developed, that is where I am happiest about. And that is really a completely different lifestyle than the one I had before. |
| 9 | P23 | Identification with fate | P23: And now I'm in this situation and I can't really do anything about it anymore.  SCer: You're a caterpillar on the bed...  P23: Yes, and that's actually driving me crazy... So it's not even necessarily 'I'm sick now and I'll never get better', but 'How on earth am I going to do it?' If I were to say now 'I'm not going to work anymore, I'm going to take the time to do fun things and rest', that's not possible at all. Because then I can't do fun things, or then the things won't be paid for anymore. So yeah, I don't know…  SCer: Yes, complicated. And that's also somewhat similar to Gregor [protagonist in *Metamorphosis*, NvP], paying off the debts, you already mentioned that. In a way, you're also stuck in... paying off the debts.  P23: Yes, there just has to be money. (...) [A]nd I think yes, of course I'm also guilty myself because I have so much stress, have had for years, an extreme amount of stress. (…) Of course I have created a situation for which I am responsible myself and for which I always take control. So to what extent do I let the other person help me? |
| 10 | P1 | Identification with fate | SCer: Why did you choose this story [*Farewell from Phoebe*]?  P1: Uhm, yes, of course it also has to do with my own loss [referring to miscarriage in the past, NvP], so to speak... And I was wondering what would be more painful, if you had a dead child or if you didn't have the possibility to get pregnant at all?  SCer: Yes, because you were about to compare it a bit?  P1: Yes.  SCer. I: And what did you think about that?  P1: I think this story is more painful, because you almost have it. But yes, at the same time I also think, yes, that I shouldn't minimize my own pain.  SCer: Yes. In a way, the loss of something you never had, is maybe more complicated..?  P1: Yes, like what they say, what memories do you have then... I don't have those either of course, not even the feeling, the joy that you are pregnant. Yes, that is something that has not happened. |
| 11 | P9 | Identification with fate | P9: [I] had a relationship with someone, we were in love for a year, very strange, madly in love, like not being able to eat or drink when the other person wasn't there. (...) But he also had slight psychopathic traits. And he was a womanizer. (...) And he suddenly thought: 'Who wants a woman with one breast? I can do whatever I want, she's not going to leave me anyway'. |
| 12 | P9 | Identification with fate | P9: And then my thoughts also go to what people have done to each other. That stuff at my work for example, what ... I worked there for 7 years after my cancer, if those guys hadn't been so mean (...) to each other, and to me and to everyone else, then I would have done much better... Then I could still be there, then I would still have had a nice job. These kinds of thoughts have been coming to me lately. |
| 13 | P1 | Identification with fate | P1: I don't really feel... there's no chaos in my life, it's... but it's also... there's also no... peace. There's just nothing. [laughing] There's no chaos. I can also sit there, like, in that emptiness, but yeah... Because some people, when they're in trouble, they feel chaos in their head. And I don't feel that. I just feel gone, nothing. (...) Being Colorless, lightless [laughing] |
| 14 | P8 | Identification with fate | P8: I think dying or being severely ill is actually something that has very little decorum left.  SC: And what do you mean by ‘decorum’?  P8: Yes, decorum… (…) You don’t work anymore, you lose your beauty, because you don’t have hair anymore. Your intestines, everything, that’s all degrading too, a stoma. Vomiting, diarrhea, everything that goes with the illness… Well, there’s actually very little left of you. |
| 15 | P4 | Experiences of normativity around behavior and emotions | P4: So that Phoebe [protagonist *Farewell from Phoebe*, NvP] (…) The essence that I see is that she sits there, the whole night with that doctor, or that doctor in training. And in fact that she tries to reassure him. He puts into words all kinds of things that she actually has to feel. And without thinking about it like ‘Yes, but how do I feel myself?’, she says like ‘Yes, but you can quietly read a book’. And then he puts into words how she has to feel. And she doesn’t protest. And I recognize that so well. |
| 16 | P24 | Experiences of normativity around behavior and emotions | P24: Yes, then you notice that people sometimes find that difficult. But the easier it is for you to talk about it yourself, yes, that opens the way for the person on the other side of the table, right? I notice that too. And our neighbors with whom we are in very good contact, but they also find it very difficult to talk about it. Well, then you avoid that, don't you? Yes, then you just talk about other things. That is sometimes annoying, then you think ‘Gosh, you can ask how it is and then I'll tell you how it is’. And yes, up until now, it still is like this. |
| 17 | P1 | Experiences of normativity around behavior and emotions | SCer: And who takes care of you then?  P1: I don't know. [laughing] Yes, I do take care of myself, but like I said, I always put someone else before me. I'm not in a situation now either, in a loving situation, like I wanted. He does support me, but I miss something, so to speak. But I can't get away, because the house is mine. He's not going to live anywhere else. Where should he live then? [laughing].  SCer: Then you also think about him, that's how you are too...  P1: Yes. (...) Yes, because he drinks a lot now, too, then I think 'Yes, if I drop out of his life, then he'll only drink even more and then’... yes. So I take care of someone else, who may not really be there for me. He takes care of me as much as he can, I think. Only the problem is, that's not enough for me. That's it. For him, he does his best, and I know that too. But you don't change a person either.  SCer: No, because, let's say, in the story [*Farewell from Phoebe*] there is also the social interaction between the main character, the mother of the child, and (…) the assistant. And yes, there is also a kind of discomfort in that, I think. That hand...  P1: Yes, from her side...  SCer: Yes, she wants to be supported.... And that hand, which she actually sees as a kind of support... But it is not support at all.  P1: No, not for her. The feeling of support is not there with her. It is more like 'Yes, I'll just do it, I'll just take that hand, because it was there the whole time anyway’. |
| 18 | P15 | Experiences of love and affection from and towards (significant) others | P15: Because like Orpheus, well I don’t make music, but the beauty of the world and of my life here and of life with my children, and my friends and family, and all the other beings that are important to me, yes I love that. (…)  SCer: Like Orpheus is interested in love, that is the answer he gives to Hades when he says: ‘How dare you come here?’ The reason is love.  P15: Yes, that is love for me too. |
| 19 | P18 | Self-regulatory aspects (exploring future scenarios) | P18: And if you... because I see a kind of scene in the forest in front of me now, where you are no longer there, you are on a journey, that is such a Toon Tellegen [author of *The Ant’s Departure*, NvP] theme. There could be a picture of an empty road and there he has left. And we are still there... He is still there, but he is on a journey, he is not coming back. And then you see those protagonists there... Then you see your children walking around with their own uniqueness and J. [wife of participant, NvP], because J. is perhaps the most problematic part, she is perhaps sitting up in the tree with her back to you or something? (...) Well, not with her back I think, but just not being able to, so to speak, carry the responsibility of two children. |
| 20 | P15 | Self-regulatory aspects (exploring impending death/articulation of existential concerns) | P15: It [metaphor] brings me self-understanding, insight into my position. I can put it into words better because I have thought about the story for a long time, mirrored in my own situation. (…) And yes, it strengthens me. So it actually brings me quite a lot, yes. (…) I see it first and I find putting it into words really much, much more difficult, but I do see it. I also see myself walking [in the underworld, NvP] [laughing]. |
| 21 | P11 | Self-regulatory aspects (exploring articulation of existential concerns) | P11: No, I don't always really feel like a beetle, not that, let's put it that way. But I can feel the fears and the helplessness of this beetle (...). That I think: oh yes, that feeling, that you really... Yes, I have had those moments, that I was lying in my bed and thought: ‘Okay, well, now you have to go to the toilet and how are you going to get out of bed, alone?’  SCer: Yes, so then it comes very close, that image?  P11: Yes, then I can really relate to that. I just would never have put it that way myself, I think, I feel like a beetle. [laughing].  SCer: And that is what is happening now in this story... And what does that do to you? Is there anything to it in comparing yourself to that?  P11: Well maybe, to be able to explain it to someone else what your feeling is. |
| 22 | P3 | Self-regulatory aspects (exploring ‘fierce’ emotions) | P3: Totally clear. This is my story [*The Ant’s Departure*]. Because, I’ll just be gone soon. And that’s exactly how I feel about it. You know, just ‘let’s go’. One day will come, and you’re just gone. And the rest just needs to cope with it. [laughing] |
| 23 | P11 | Self-regulatory aspects (exploring ‘fierce’ emotions/existential concerns) | P11: Well, that's the part that triggers me to choose this story [*Metamorphosis*] (...) And of course, not with the crazy legs in the air, I can't even get them in the air anymore... [laughing], but of course, the hopeless feeling that you have then. And of course people shout, and you say as best you can: 'No, nothing's wrong, I'll be there in a minute!' But actually you think: 'My goodness, how am I going to get out of here?' |
| 24 | P22 | Self-regulatory aspects (exploring ‘fierce’ emotions) | P22: I hope to be able to hold on for a while, without much pain, because I think that... Lately, I've been suffering more from physical complaints, I think I shouldn't suffer too much from that. Then, I'd rather have that pill or that injection. Yes. [laughing] |
| 25 | P22 | Self-regulatory aspects (exploring ‘fierce’ emotions/impending death) | P22: Yes, I really feel like I'm moving forward. And I realized that when I read this story. I thought ‘Oh yeah... those emotions [sadness and grief, NvP] haven't been there much yet, that intensity.’ (...) It has broken down a barrier, let's say, in my emotions, this story. I've read a lot lately, often to keep it [thinking about death, NvP] at a distance. And maybe because we're in this process [of participating in the project ISOS, NvP], that I'm more aware of the fact (...) that I belong to that group where it's just not there anymore, that there's no longer any prospect of recovery. That's slowly starting to sink down on me. (...) Yes, you know, you've reached the point where you no longer want to deny that it's there. |
| 26 | P22 | Self-regulatory aspects (exploring impending death) | P22: Yes, I also notice that I am thinking more about the final process: do I want to go to a hospice, or do I want to be cared for at home? How do I want to be... buried or cremated... You know, I notice that I'm shifting more in that. Which I've actually avoided quite a bit up until now. Because that just suits me, being a bit avoidant, rational, but I'm more concerned with that now. Yes.  SCer: Grandpa [protagonist *Code Catnip*NvP] is also avoidant, doesn't want to do it either, and is also rational and says 'Yes, I'm going to die', in that sense, he's quite harsh...  P22: But not yet...  SCer: Exactly. When the time comes, I'll decide for myself. But you see, the family imposes it, as it were...  P22: Yes, I mean, and that is also the response that one develops, when people then, so to speak... There was a period when I had bad news, that there was another metastasis, that people thought it was all very terrible, and then I was rather rational... 'Oh, I'm not dead yet, it will come... and I can hold on for a while', you know, like that. I recognize that response. I mean, the more others suffer from it, the more they talk about it, the less I talk about it. And now, I am slowly turning this around. |
| 27 | P26 | Self-regulatory aspects (broadening perspectives on the self) | P26: Well, if I... What is a comforting thought for me, in this story, is that ant [protagonist *The Ant’s Departure*, NvP] actually didn’t do that much to leave his legacy... Making sure that he built incredibly beautiful buildings, wrote wonderful books... He only sent a note, ‘The ant, that’s me.’ That was for himself. And the other one was ‘Bye rhino, the ant.’ So actually, just by being there, that’s enough. I sometimes get overwhelmed by an enormous pressure of ‘I still have things to do, I still have things to leave behind, I still have to fulfil my moral duty on earth.’ At the moment, I’m passed that thought. I also joined the board of a football club, and then you notice... And of all kinds of professional associations at work, I also want this and that for my children... It’s also good to realize that if it were over now, that would be fine too. Then I think I would have... Then they would have enough memories, too. That doesn't depend on what you do in this final period of life. |
| 28 | P4 | Self-regulatory aspects (strengthening identity) | P4: Well that was (…) back in 2016, I was so far gone… Then he [P4’s husband, NvP] did it himself. He went to people [healthcare professionals, NvP] in the hallway, like ‘Listen to me!’. So they were, yeah, I wasn’t getting any nutrients, I wasn’t eating anymore… So I was dehydrated. And so he [husband] insisted that I got those special food bags. So I got that astronaut food. And that did make me feel better. (…) But I wouldn’t do that. And that Otto [protagonist *Farewell from Phoebe*, who is avoiding taking sincere care of his wife, NvP], if he had been [my husband] he wouldn't have gone home either, he would have stayed there and taken a nap. And that's also the story...  SCer: And how do you understand that for yourself, that you actually only want to be liked, to go along with what other people want from you, that it could even mean the end of your life?  P4: It's so deep. Well, maybe, if we go back to that story, it's so deep inside of me. So, participating, belonging to a group, not being excluded from the group. Look, what I'm saying, I've gotten better. You don't want to know how far I went to avoid getting into a conflict. Well, I've learned that, some conflicts you just have to accept. |
